# Effects of interval versus continuous exercise on cerebral vascular flow‐mediated dilatation in young healthy males

**DOI:** 10.14814/phy2.70354

**Published:** 2025-05-05

**Authors:** Harvey J. Walsh, Shotaro Saito, Narumi Kunimatsu, Marino Karaki, James P. Fisher, Shigehiko Ogoh

**Affiliations:** ^1^ Department of Physiology Faculty of Medical & Health Sciences, University of Auckland Auckland New Zealand; ^2^ Department of Biomedical Engineering Toyo University Kawagoe Japan; ^3^ Neurovascular Research Laboratory University of South Wales Pontypridd UK

**Keywords:** cerebrovascular, endothelium, exercise, humans, ultrasound

## Abstract

Aerobic exercise reduces the risk of cerebrovascular dysfunction. One proposed mechanism is exercise‐induced increases in cerebral shear stress (SS) improving cerebral endothelial function. A recent report indicated that interval exercise (Int‐Ex) induces greater cerebral SS than continuous exercise (Con‐Ex); however, its effect on cerebral endothelial function remains unclear. We hypothesized that Int‐Ex would enhance cerebral SS and, consequently, cerebral endothelial function more than Con‐Ex. Fourteen healthy males (21 ± 0.6 years) completed 32 min of Int‐Ex and work‐equivalent Con‐Ex on a semi‐recumbent bike on separate days. Cerebrovascular flow‐mediated dilatation (cFMD) was assessed before exercise (Pre), 15 min (Post‐15) and 40 min post‐exercise (Post‐40). cFMD was defined as peak internal carotid artery vasodilatation (Δ% from baseline; Duplex ultrasound) in response to a 30‐s hypercapnic exposure, raising end‐tidal partial pressure of carbon dioxide by ~7 mmHg. Post‐exercise cerebral SS was greater after Int‐Ex versus Con‐Ex (*p* = 0.002). Int‐Ex evoked a ~37% increase in post‐exercise cerebral SS compared to rest, with a negligible increase for Con‐Ex. cFMD did not differ between Int‐Ex and Con‐Ex trials before exercise (Pre, 6.35 ± 3.89% vs. 5.54 ± 3.83%; *p* = 0.542) and remained unchanged post‐exercise (Post‐15, 7.20 ± 4.47% vs. 6.13 ± 4.08%; Post‐40, 5.69 ± 3.86% vs. 6.94 ± 3.55%; *p* = 0.583). These results indicate that Int‐Ex and Con‐Ex have similar acute effects on cerebral endothelial function.

## 1 INTRODUCTION

Aerobic exercise has been reported to mitigate age‐related neurodegeneration and deficits in cerebrovascular function (Zimmerman et al., 2021) by helping maintain adequate cerebral perfusion (Kleinloog et al., 2019). Although the precise mechanisms underlying these exercise‐induced improvements remain unclear, enhanced cerebral endothelial function is one potential mechanism (Ainslie et al., 2008; Anazodo et al., 2015; Bliss et al., 2021; Joyner & Green, 2009; Sakamoto et al., 2024). Exercise‐induced increases in shear stress (SS), defined as the mechanical interaction between the vessel wall and luminal blood flow, have been shown to enhance endothelial function and nitric oxide (NO) bioavailability (Davies, 2009), both of which are linked to improved cardiovascular health (Pinckard et al., 2019). While this relationship is well established in the conduit arteries of the peripheral (Desouza et al., 2000) and coronary circulations (Duncker & Bache, 2008), the influence of blood flow patterns and SS on cerebral endothelial function remains not fully understood. Importantly, SS in the cerebral circulation is entirely different from that in the peripheral circulation due to the absence of retrograde flow in the cerebral circulation (Rodrigues et al., 2020). Clarifying this relationship is crucial for developing exercise modalities that most effectively improve cerebrovascular function and brain health in relevant populations.

Sakamoto and colleagues recently demonstrated that moderate‐intensity aerobic exercise increased SS within the internal carotid artery (ICA) and enhanced cerebrovascular flow‐mediated dilatation (cFMD), an in vivo bioassay of cerebral vascular responsiveness primarily mediated by NO (Hoiland et al., 2022; Sakamoto et al., 2024). Notably, the magnitude of cerebral SS is influenced by the mode of aerobic exercise. Ogoh et al. (2021) observed that interval aerobic exercise (Int‐Ex), characterized by varying exercise intensity, induces greater cerebral SS compared to work‐equivalent continuous aerobic exercise (Con‐Ex). However, it remains unknown whether these differences in cerebral hemodynamics impact the effect of exercise on cerebrovascular endothelial function. Therefore, the aim of this study was to test the hypothesis that Int‐Ex evokes greater SS and greater improvements in cFMD, a measure of cerebral endothelial function, compared to work‐equivalent Con‐Ex.

## 2 MATERIALS AND METHODS

### 2.1 Ethical approval

The experimental protocol was approved by the Human Subjects Committee of Toyo University (TU2023‐37). Study participants received an information sheet outlining all experimental procedures, and any questions were addressed prior to providing informed written consent. All study procedures conformed to the standards set by the *Declaration of Helsinki*, excluding study registration in a database.

### 2.2 Participant characteristics

Fourteen healthy adult males (age: 21.4 ± 0.6 years; height: 1.72 ± 0.05 m; weight: 62.8 ± 6.6 kg; body mass index: 21.5 ± 2.2 kg/m^2^. Mean ± standard deviation [SD]) were included in this study. Participants had no overt cerebrovascular, cardiovascular, neurological, metabolic, or pulmonary disease, were not taking any prescribed or over‐the‐counter medications, were non‐smokers, and were not abusers of alcohol. In addition, participants reported that they participated in <5 h/week of regular aerobic exercise. Before each experimental visit, participants were requested to abstain from caffeine consumption for 12 h, and from alcohol and strenuous exercise for 24 h.

### 2.3 Experimental measures

#### 2.3.1 Cardiorespiratory

Beat‐to‐beat arterial blood pressure (ABP) was continuously monitored by finger plethysmography from a cuff around the middle finger of the right hand (Finapres Medical Systems, Amsterdam, Netherlands). Non‐invasive brachial artery blood pressure (BP) was measured using an automated digital cuff sphygmomanometer and used to calibrate beat‐to‐beat ABP (Tango +; SunTech Medical, Eynsham (Witney), UK). Heart rate (HR) was measured using a lead II electrocardiogram (ECG) (Bedside monitor, BMS‐3400; Nihon Kohden, Tokyo, Japan). Participants wore an oronasal mask connected to an automated gas analyzer (AE‐310S; Minato Medical Science, Osaka, Japan) to measure minute ventilation (V̇_E_), respiratory rate (RR) and end‐tidal partial pressure of carbon dioxide (P_ET_CO_2_). Analogue signals were recorded and digitized at 1 KHz using a multi‐channel data acquisition system (PowerLab16s; ADinstruments, Sydney, Australia), and data was stored and analyzed on a computer offline.

#### 2.3.2 Cerebrovascular

The mean blood velocity and diameter of the right ICA were measured using duplex Doppler ultrasound (Vivid I; GE Healthcare, Chicago, IL, USA) equipped with a 13‐MHz linear transducer. Imaging of the right ICA was performed 1–1.5 cm cranial to the carotid bifurcation. Brightness (B) and pulsed‐wave modes were used to obtain diameter and mean blood velocity measures, and a constant insonation angle (<60°) was ensured. A video capture device (DVI2USB 3.0; Epiphan Systems, Ottawa, Canada) was used to record cerebrovascular images at 30 Hz as a video file ready for offline analysis.

#### 2.3.3 Cerebrovascular flow mediated dilation (cFMD)

A transient hypercapnic exposure (30 s) was used to characterize cerebral endothelial function, as previously reported (Hoiland et al., 2022). CO_2_‐induced shear‐mediated ICA vasodilatation was assessed by measurement of right ICA diameter for 5 min 30 s (2 min‐baseline, 30 s‐transient hypercapnic exposure, 3‐min post‐hypercapnic). Mixed gas (room air and 100% CO_2_) was delivered to the participant during the transient hypercapnic exposure via a mixing chamber (250 mL gas blender; Arco System, Chiba, Japan). The CO_2_ concentration in the mixing chamber was manually adjusted to ensure a quick elevation (~5 s) in P_ET_CO_2_ from baseline by ~7 mmHg. A metronome was used to pace participants at 20 breaths/minute, as previously described (Hoiland et al., 2017, 2022). This higher breathing rate was used to ensure quick gas manipulations and keep minute ventilation (V̇_E_) constant. In addition, visual feedback was used to perform fine adjustments to tidal volume (V_T_).

### 2.4 Experimental protocol

Each participant completed two exercise trials (i.e., continuous aerobic exercise or work‐equivalent interval aerobic exercise, 153.6 KJ/exercise session) on separate days with trial order randomly assigned by coin toss (Ogoh et al., 2021). Recumbent leg cycling (Aerobike 75XL III, Combi) was performed at 60 rpm, with the participants' left arm placed on an examination table and their right arm extended vertically by their side to maintain stable BP measurements. A metronome was used to pace participants at the target rpm, which was checked throughout the exercise period. Exercise onset was preceded by a 2‐min rest period and a standardized warm‐up of 2 min of cycling at 20 Watts (W). Continuous exercise was performed at 80 W for 32 min (work rate: 80 W × (32 × 60 s) = 153.6 KJ). Interval exercise was performed for 32 min and included eight rounds of interval cycling, each including 2 min at 60 W and 2 min at 100 W (work rate: 60 W × (2 × 60 s) + 100 W × (2 × 60 s) × 8 rounds = 153.6 KJ). These exercise intensities were used as we have previously demonstrated that they evoke differential haemodynamic responses in the extracranial vessels (Ogoh et al., 2021). A 2‐min recovery period was included at the conclusion of exercise. A cFMD test was performed prior to exercise and at the 15th and 40th minutes post‐exercise.

### 2.5 Data analysis

The ABP waveform was used to obtain beat‐to‐beat systolic and diastolic BP. Mean arterial pressure (MAP) was obtained beat‐to‐beat by integration of the ABP waveform over the complete cardiac cycle. HR was calculated extracted beat‐to‐beat from the ECG. P_ET_CO_2_ and V̇_E_ were measured breath‐by‐breath. All variables underwent offline analysis (LabChart 8, ADInstruments). Ultrasound video files were processed with custom‐designed edge‐detecting and wall‐tracking software (version 2.0.4, S‐13037; Takei Scientific Instruments, Niigata, Japan) to determine ICA diameter and mean blood velocity. Data interpolation was performed where ICA diameter data was missing using the least squares interpolation method. This was followed by data resampling to 1 Hz and data filtering using a two‐stage filtering process including a Savitzky–Golay finite impulse response smoothing filter and a median filter. The ICA shear rate (SR) was calculated as SR (/s) = 4 × blood velocity/diameter. ICA blood flow was calculated as blood flow (mL/min) = π × (diameter/2)^2^ × blood velocity × 60. ICA cerebrovascular conductance index (CVCi) (mL/min/mmHg) was calculated as the ratio of blood flow with MAP.

Across the exercise period, cardiorespiratory and cerebrovascular data were averaged across the final 30 s of rest and the entire 2‐min recovery. In addition, the final 4 min of exercise was averaged for cardiorespiratory data. For each cFMD test, data was analyzed as previously reported by Saito et al. (2023). Data was averaged across the 2‐min baseline, 30‐s transient hypercapnic exposure, and 3‐min post‐transient hypercapnic exposure. More specifically, ICA baseline diameter and ICA baseline SR were defined as the median ICA diameter and median ICA SR values during the 2‐min baseline, respectively. ICA peak diameter and ICA SR values were identified during the 3‐min post‐transient hypercapnic exposure. cFMD was calculated as cFMD (%) = (ICA peak diameter − ICA baseline diameter)/ICA baseline diameter × 100.

Visual inspection of ultrasound videos ensured adequate data quality, and trials were excluded on the basis of excessive vessel movement (due to high ventilation), poor wall tracking (despite high image quality) and overall poor image quality where wall tracking could not be accurately completed. Four trials were excluded from the analysis of the exercise protocol, and nine trials were excluded from the cFMD tests. Sample sizes are included in table notes and figure legends for reference.

### 2.6 Statistical analysis

Data normality was assessed by visual inspection of histograms and QQ‐plots. All data are presented as mean ± standard deviation (SD). Linear mixed model analysis was used to detect the effect of time (rest and exercise) and exercise modality (Con‐Ex and Int‐Ex) on cardiorespiratory data during the exercise protocol (factors: Time × Exercise modality). The same analysis was used for cerebrovascular data, except only Rest and Recovery were included for the effect of time. Similarly, linear mixed model analysis was used to detect the effect of time (Pre, Post‐15 and Post‐40), transient hypercapnia (Baseline and Transient hypercapnia) and exercise modality (Con‐Ex and Int‐Ex) on cardiorespiratory data during cFMD assessment (factors: Time × Transient hypercapnia × Exercise modality). Cerebrovascular data during cFMD assessment was analyzed by linear mixed model analysis to detect the effect of time (Pre, Post‐15 and Post‐40) and exercise modality (Con‐Ex and Int‐Ex) (factors: Time × Exercise modality). The same model was used to assess corrected‐cFMD, but with the addition of D_base_ and SR_AUC_ as covariates. Upon identification of significant main effects or interactions, post‐hoc pairwise comparisons were performed using Student's *t*‐tests with Bonferroni correction. Statistical significance was considered as *p* < 0.05. Statistical analysis was performed using SPSS, version 27 (IBM Corp., Armonk, NY, USA).

## 3 RESULTS

### 3.1 Cardiorespiratory and cerebrovascular responses to exercise

Table 1 displays cardiorespiratory data during rest, exercise, and recovery. Compared with rest, DBP, MAP, HR, P_ET_CO_2_, V_E_, and RR were elevated during exercise and recovery (all *p* < 0.05). In addition, HR, V_E_, and RR were lower during recovery compared to exercise (all *p* < 0.001). The magnitude of the exercise‐induced increase in SBP was greater with Con‐Ex compared to Int‐Ex (*p* = 0.031), and SBP was reduced during recovery after Con‐Ex (*p* < 0.001) but not after Int‐Ex (*p* = 1.000).

**TABLE 1:** Cardiorespiratory data at rest, during exercise and recovery.

	Con‐Ex			Int‐Ex			*p‐*Value		
	Rest	Exercise	Recovery	Rest	Exercise	Recovery	Time	Exercise modality	Interaction
SBP (mmHg)	134.9 ± 14.7	166.8 ± 21.1*	150.6 ± 15.5*^,#^	129.2 ± 15.5	158.5 ± 17.1*^,†^	156.0 ± 11.9*	<0.001	0.195	0.030
DBP (mmHg)	81.9 ± 9.0	90.1 ± 15.0	87.8 ± 11.5	79.8 ± 14.5	87.9 ± 10.8	88.3 ± 8.1	<0.001^a,b^	0.466	0.764
MAP (mmHg)	99.4 ± 9.5	115.3 ± 15.2	108.6 ± 12.4	96.5 ± 13.9	111.3 ± 10.2	110.9 ± 8.5	<0.001^a,b^	0.300	0.176
HR (beat/min)	75.6 ± 15.3	135.8 ± 15.8	110.9 ± 19.0	77.8 ± 7.8	139.2 ± 17.7	117.0 ± 20.3	<0.001^a–c^	0.051	0.702
P_ET_CO_2_ (mmHg)	37.8 ± 3.5	43.1 ± 3.8	41.0 ± 2.9	38.3 ± 2.8	42.9 ± 2.3	42.3 ± 2.5	<0.001^a,b^	0.234	0.435
V_E_ (L/min)	10.4 ± 1.5	37.9 ± 3.3	22.2 ± 3.6	11.1 ± 1.5	40.3 ± 3.6	24.9 ± 4.0	<0.001^a–c^	<0.001	0.258
RR (breath/min)	17.7 ± 3.6	29.4 ± 4.6	21.0 ± 4.1	19.2 ± 4.2	32.6 ± 4.6	22.6 ± 4.9	<0.001^a–c^	0.001	0.454

*Note*: Upon identification of a significant interaction, differences observed through post‐hoc analysis (*t*‐tests with Bonferroni correction) are displayed as *p < 0.05 different from Rest, ^#^ *p* < 0.05 different from Exercise, ^†^ *p* < 0.05 different from Con‐Ex. Upon identification of a significant main effect of time, but no interaction, differences observed through post‐hoc analysis (*t*‐tests with Bonferroni correction) are displayed as ^a^ *p* <0.05 Rest versus Exercise, ^b^ *p* <0.05 Rest versus Recovery, ^c^ *p* <0.05 Exercise versus Recovery. Cardiorespiratory data: *n* = 14.

Abbreviations: Con‐Ex, continuous exercise; DBP, diastolic blood pressure; HR, heart rate; Int‐Ex, interval exercise; MAP, mean arterial pressure; P_ET_CO_2_, end‐tidal partial pressure of CO_2_; RR, respiratory rate; V_E_, minute ventilation; SBP, systolic blood pressure.

**FIGURE 1:**
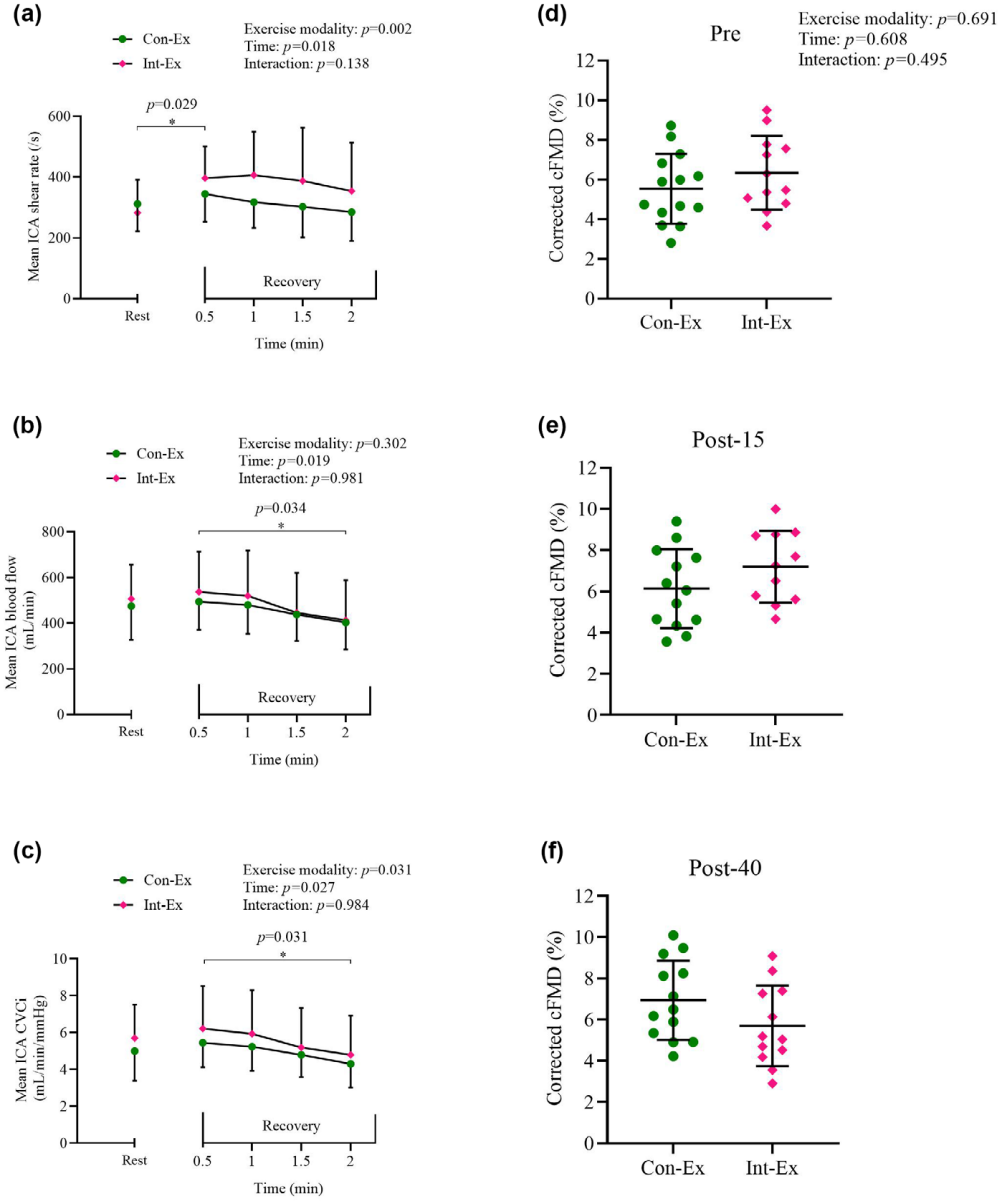
(a–c) ICA haemodynamics evoked by exercise. (d–f) Corrected‐cFMD before and after exercise interventions. (a) Mean ICA shear rate, (b) mean ICA blood flow and (c) mean ICA CVCi at rest and during 2 min recovery after Con‐Ex and Int‐Ex. Data was averaged across the final 30 s of rest and across the entire 2 min recovery period. Data presented as mean ± SD. Upon identification of a significant main effect of time, but no interaction, differences observed through post‐hoc analysis (t‐tests with Bonferroni correction) are displayed with *p*‐values. Cerebrovascular data: *N* = 14 for Con‐Ex Recovery, *n* = 13 for Con‐Ex Rest and Int‐Ex Recovery, *n* = 12 for Int‐Ex Rest. (d) Corrected‐cFMD before (Pre), (e) 15‐min (Post‐15) and (f) 40‐min (Post‐40) after Con‐Ex or Int‐Ex. cFMD values were corrected for SR_AUC_ and D_base_. Individual values presented as median. Bars display mean ± SD. ICA data: *N* = 14 for Continuous Pre, *n* = 13 for Continuous Post‐15 and Post‐40, *n* = 12 for Interval Pre and Post‐40, *n* = 11 for Interval Post‐15. cFMD, cerebrovascular flow‐mediated dilatation; Con‐Ex, continuous exercise; CVCi, cerebrovascular conductance index; D_base_, baseline diameter; ICA, internal carotid artery; Int‐Ex, interval exercise; SR_AUC_, shear rate area under the curve.

Cerebrovascular data during the rest and recovery periods of the exercise protocol are displayed in Figure 1a–c. Mean ICA shear rate and mean ICA CVCi were greater in Int‐Ex compared to Con‐Ex, while mean ICA blood flow was not different between exercise modalities. In addition, the mean ICA shear rate was higher at the 0.5‐min recovery compared to rest (*p* = 0.029), while the mean ICA blood flow (*p* = 0.034) and mean ICA CVCi (*p* = 0.031) were reduced at the 2‐min recovery compared to the 0.5‐min recovery.

### 3.2 cFMD assessment

Cardiorespiratory data during the cFMD assessment are displayed in Table 2. Compared with pre, SBP and MAP (*p* = 0.025) were lower at post‐40, whilst HR was higher at post‐15 (*p* < 0.001). In addition, SBP (*p* = 0.017) and HR (*p* = 0.005) were lower at post‐40 compared to post‐15. Exercise modality impacted DBP, MAP, and V_E_, with lower DBP and MAP in Int‐Ex and lower V_E_ in Con‐Ex. Transient hypercapnia evoked increases in SBP, HR, and P_ET_CO_2_ as compared to baseline (Table 2; *p* = 0.011, *p* = 0.006, *p* < 0.001). An interaction was detected between Exercise modality and Transient hypercapnia for P_ET_CO_2_. Post‐hoc analysis indicated increased P_ET_CO_2_ during transient hypercapnia for both Con‐Ex and Int‐Ex (both *p* < 0.001) and no difference in P_ET_CO_2_ between Con‐Ex and Int‐Ex at either baseline (*p* = 0.051) or during transient hypercapnia (*p* = 0.237).

**TABLE 2:** Cardiorespiratory data during cerebrovascular flow‐mediated dilation testing.

	Exercise modality	Pre		Post 15		Post 40		*p*‐Value		
		Baseline	Transient hypercapnia	Baseline	Transient hypercapnia	Baseline	Transient hypercapnia	Time	Exercise modality	Transient hypercapnia
SBP (mmHg)	Con‐Ex	121.4 ± 12.9	126.6 ± 16.3	121.0 ± 12.9	125.0 ± 14.8	120.3 ± 14.5	120.4 ± 15.2	0.001^b,c^	0.045	0.011
	Int‐Ex	120.7 ± 12.3	126.0 ± 14.9	120.1 ± 12.2	124.4 ± 13.7	118.3 ± 12.4	118.0 ± 13.5			
DBP (mmHg)	Con‐Ex	72.0 ± 8.9	74.4 ± 10.0	69.1 ± 14.0	71.1 ± 14.3	70.0 ± 12.2	70.1 ± 12.2	0.188	<0.001	0.230
	Int‐Ex	65.8 ± 8.4	68.2 ± 9.2	67.7 ± 10.0	69.5 ± 11.1	65.1 ± 9.9	64.9 ± 10.0			
MAP (mmHg)	Con‐Ex	88.4 ± 9.5	91.5 ± 11.1	86.2 ± 12.5	88.5 ± 12.9	86.6 ± 12.6	86.8 ± 12.7	0.025^b^	<0.001	0.073
	Int‐Ex	83.8 ± 8.6	86.7 ± 9.5	84.9 ± 9.1	87.1 ± 10.5	82.1 ± 7.9	82.1 ± 8.4			
HR (beat/min)	Con‐Ex	71.8 ± 14.3	75.3 ± 14.6	75.9 ± 15.2	79.1 ± 15.0	74.0 ± 14.4	75.7 ± 15.0	<0.001^a,c^	0.534	0.006
	Int‐Ex	73.0 ± 11.4	76.0 ± 10.8	77.8 ± 12.2	79.1 ± 11.5	74.0 ± 9.7	75.1 ± 9.7			
P_ET_CO_2_ (mmHg)	Con‐Ex	39.4 ± 3.4	46.7 ± 3.3	38.7 ± 3.5	46.5 ± 3.3	38.4 ± 3.4	46.2 ± 3.7	0.105	0.582*	<0.001*
	Int‐Ex	40.3 ± 4.4	46.6 ± 5.0	39.3 ± 3.8	45.6 ± 3.8	39.6 ± 4.1	45.6 ± 4.0			
V_E_ (L/min)	Con‐Ex	10.5 ± 1.9	10.3 ± 2.6	10.6 ± 1.8	10.5 ± 1.8	10.1 ± 1.8	9.8 ± 2.0	0.533	<0.001	0.797
	Int‐Ex	11.7 ± 1.6	11.5 ± 2.1	12.0 ± 2.7	11.7 ± 2.7	11.4 ± 2.3	12.1 ± 2.7			
RR (breath/min)	Con‐Ex	20.0 ± 0.4	20.8 ± 2.7	20.2 ± 0.2	20.3 ± 0.5	20.1 ± 0.3	20.2 ± 0.4	0.519	0.050	0.445
	Int‐Ex	20.1 ± 0.2	19.9 ± 0.6	20.0 ± 0.3	19.9 ± 0.5	20.1 ± 0.1	19.9 ± 0.4			

*Note*: Data presented as mean ± SD. Upon identification of a significant main effect of time, but no interaction, differences observed through post‐hoc analysis (t‐tests with Bonferroni correction) are displayed as ^a^ *p* <0.05 Pre versus Post‐15, ^b^ *p* <0.05 Pre versus Post‐40, ^c^ *p* <0.05 Post‐15 versus Post‐40. A significant interaction was only observed for Exercise modality × Transient hypercapnia (*p < 0.005). Post‐hoc analysis (*t*‐tests with Bonferroni correction) revealed *p* < 0.05 Baseline versus Transient hypercapnia for both Con‐Ex and Int‐Ex. Cardiorespiratory data: *n* = 14.

Abbreviations: DBP, diastolic blood pressure; HR, heart rate; MAP, mean arterial pressure; P_ET_CO_2_, end‐tidal partial pressure of CO_2_; RR, respiratory rate; V_E_, minute ventilation; SBP, systolic blood pressure.

Table 3 displays cerebrovascular data during cFMD testing. SR_base_ and SR_peak_ were lower at post‐40 compared to pre (both *p* < 0.001) and post‐15 (*p* = 0.011, *p* = 0.013), whilst blood flow peak was lower at post‐15 compared to pre (*p* = 0.045). No differences were observed in D_base_, D_peak_, time to peak dilatation, SR_AUC_, blood flow base, CVCi_base_, and CVCi_peak_ across time or exercise modalities (all *p* > 0.05). Cerebrovascular flow‐mediated dilatation and corrected‐cFMD (Figure 1d–f) were not different at pre, post‐15, and post‐30 time points (*p* = 0.584, *p* = 0.608) and between the Con‐Ex and Int‐Ex exercise modality (*p* = 0.583, *p* = 0.691).

**TABLE 3:** Internal carotid artery responses during cerebrovascular flow‐mediated dilation testing.

	Con‐Ex			Int‐Ex			*p‐*Value		
	Pre	Post‐15	Post‐40	Pre	Post‐15	Post‐40	Time	Exercise modality	Interaction
D_base_ (mm)	4.9 ± 0.5	4.7 ± 0.5	5.1 ± 0.7	5.1 ± 1.0	5.0 ± 1.0	5.2 ± 0.9	0.112	0.222	0.436
D_peak_ (mm)	5.2 ± 0.6	5.00 ± 0.6	5.5 ± 0.8	5.5 ± 1.2	5.4 ± 1.1	5.5 ± 1.1	0.129	0.142	0.249
Time to peak dilatation (s)	110.6 ± 56.2	129.2 ± 55.1	112.9 ± 43.2	100.9 ± 47.3	119.5 ± 57.7	120.8 ± 51.7	0.453	0.748	0.790
SR_base_ (/s)	366.7 ± 68.1	363.7 ± 73.2	304.1 ± 69.7	359.0 ± 113.8	337.2 ± 101.6	285.8 ± 79.1	<0.001^b,c^	0.392	0.812
SR_peak_ (/s)	463.3 ± 87.1	444.3 ± 86.7	371.5 ± 94.6	431.5 ± 138.9	401.5 ± 140.7	343.5 ± 95.0	<0.001^b,c^	0.149	0.902
SR_AUC_ (a.u.)	30,272 ± 19,846	30,269 ± 15,390	20,788 ± 10,379	24,465 ± 16,406	21,669 ± 8717	23,685 ± 18,161	0.442	0.269	0.321
cFMD (%)	5.5 ± 3.8	6.1 ± 4.1	6.9 ± 3.5	6.3 ± 3.9	7.2 ± 4.5	5.7 ± 3.9	0.584	0.583	0.394
Blood flow_base_ (mL/min)	532.7 ± 181.6	447.5 ± 136.4	477.9 ± 171.9	565.0 ± 241.0	503.6 ± 231.0	473.9 ± 234.6	0.077	0.637	0.571
Blood flow_peak_ (mL/min)	688.2 ± 215.9	563.9 ± 170.5	621.8 ± 216.9	723.3 ± 290.9	625.2 ± 263.6	601.3 ± 297.9	0.024^a^	0.709	0.453
CVCi_base_ (mL/min/mmHg)	6.1 ± 2.1	5.2 ± 1.3	5.5 ± 1.8	6.6 ± 2.6	6.0 ± 2.9	5.7 ± 2.9	0.184	0.277	0.686
CVCi_peak_ (mL/min/mmHg)	7.8 ± 2.5	7.0 ± 2.0	8.1 ± 2.8	8.7 ± 3.5	7.6 ± 3.4	7.6 ± 3.7	0.268	0.502	0.331

*Note*: Data presented as mean ± SD. Upon identification of a significant main effect of time, but no interaction, differences observed through post‐hoc analysis (*t*‐tests with Bonferroni correction) are displayed as ^a^ *p* <0.05 Pre versus Post‐15, ^b^ *p* <0.05 Pre versus Post‐40, ^c^ *p* <0.05 Post‐15 versus Post‐40. ICA data: *n* = 14 for Con‐Ex Pre, *n* = 13 for Con‐Ex Post‐15 and Post‐40, *n* = 12 for Int‐Ex Pre and Post‐40, *n* = 11 for Int‐Ex Post‐15.

Abbreviations: Blood flow_base_, baseline blood flow; Blood flow_peak_, peak blood flow; cFMD, cerebrovascular flow‐mediated dilatation; Con‐Ex, continuous exercise; CVCi_base_, baseline cerebrovascular conductance index; CVCi_peak_, peak cerebrovascular conductance index; D_base_, baseline diameter; D_peak_, peak diameter; Int‐Ex, interval exercise; Pre, before exercise intervention; Post‐15, 15 min after exercise intervention; Post‐40, 40 min after exercise intervention; SR_AUC_, shear rate area under the curve; SR_base_, baseline shear rate; SR_peak_, peak shear rate.

## 4 DISCUSSION

The aim of this study was to elucidate the effects of Int‐Ex and Con‐Ex on cerebral SS and cerebral vascular endothelial function. This study found that, despite differences in post‐exercise cerebral SS, there was no difference in cerebral vascular endothelial function between Int‐Ex and Con‐Ex.

Healthy aging is associated with pronounced neurodegeneration and deficits in cerebrovascular function, leading to cognitive dysfunction across multiple domains (e.g., executive function, working memory, attentional tasks) (Murman, 2015) and increasing the risk of brain diseases such as dementia. Thus, understanding how different exercise modalities may mitigate these effects is crucial for informed exercise prescriptions. In the intracranial arteries, Int‐Ex has demonstrated greater accumulated changes in middle cerebral artery blood velocity compared to Con‐Ex (Klein et al., 2019), often characterized by a rebounding effect whereby blood velocity measures from transcranial Doppler increase during recovery periods (Labrecque et al., 2020; Weaver et al., 2021; Whitaker et al., 2022). Our previous work has shown that Int‐Ex and Con‐Ex evoke different cerebral hemodynamic profiles in the extracranial arteries (Ogoh et al., 2021), both during and post‐exercise. Consistent with these findings, the present study demonstrated that post‐exercise cerebral SS responses were greater after Int‐Ex compared to Con‐Ex. Since our two exercise conditions were dose‐matched and produced similar changes in P_ET_CO_2_ across the exercise trials (Table 1), differential changes in P_ET_CO_2_ were unlikely to be the primary driver of the differences in cerebral SS responses between exercise modalities. The exact mechanisms underpinning these differences remain incompletely understood but may be related to the specific SS phenotype evoked by different exercise modalities within the cerebrovasculature (Ogoh et al., 2021; Rodrigues et al., 2020).

Despite Int‐Ex eliciting a greater increase in post‐exercise cerebral SS compared to Con‐Ex, cerebral endothelial function remained unchanged at both post‐exercise time points. The lack of improvement in cFMD may be attributed to the elevation in sympathetic nervous activity induced by our exercise protocol (Dawson et al., 2013). In the cerebral vasculature, ICA dilatation in response to hypercapnia has been shown to be attenuated by heightened sympathetic activity resulting from high‐intensity exercise (Sakamoto et al., 2021). Interestingly, this phenomenon is also observed in reduced brachial artery flow‐mediated dilatation (FMD), which remains diminished up to sixty minutes post‐exercise, whereby it returns to baseline levels (Birk et al., 2013). Notably, α_1_‐adrenoreceptor blockade has been shown to negate this negative effect on brachial FMD immediately post‐exercise, indicating a direct sympathetic vasoconstrictor influence (Atkinson et al., 2015). Thus, sympathetic‐mediated vasoconstriction may have counteracted ICA NO‐mediated vasodilatation, thereby attenuating improvements in cFMD. Furthermore, the exercise workload in the present study was relatively lower compared to typical exercise training, which may have diminished the potential benefits of Int‐Ex. Additionally, a lower workload could contribute to increased individual variability. Notably, this study employed an absolute rather than a relative workload, which may have further influenced the observed post‐exercise endothelial function.

Certain experimental limitations are associated with the findings of this study. Firstly, we only included adult males, limiting our ability to assess sex‐specific differences in the effects of the two exercise modes on cerebral SS and cFMD. The authors recognize the importance of including both male and female participants in human physiology studies. The present study was advertised to both males and females attending the Toyo University Kawagoe campus; however, the campus undergraduate population is >80% male. Secondly, our participant cohort included only young healthy adults. The cardiovascular and cerebrovascular effects of exercise in older healthy adults and clinical populations are known to be different from those of young healthy adults; thus, we cannot directly generalize our results to these demographics. Thirdly, previous work by Sakamoto et al. demonstrated improvements in cFMD 10 min post exercise (Sakamoto et al., 2024), indicating an immediate response from the cerebral endothelium. However, we acknowledge our limited ability to observe potential later changes in cerebral endothelial function after our final cFMD measurement at 40 min post‐exercise. Finally, we utilized a 3‐factor linear mixed model for analysis of our cardiorespiratory measures in Table 2, and we acknowledge the heightened risk of type II error associated with our sample size.

In conclusion, despite eliciting different post‐exercise cerebral hemodynamic responses, Int‐Ex and Con‐Ex appear to have similar acute effects on cerebral endothelial function. Further studies are needed to better understand how to optimize exercise to improve brain health.

## AUTHOR CONTRIBUTIONS

JPF and SO conceived and designed the research; HJW, SS, NK, and MK performed experiments; HJW and SS analyzed data; HJW, SS, JPF, and SO interpreted the results of experiments; HJW prepared figures; HJW drafted the manuscript; HJW, JPF, and SO edited and revised the manuscript; HJW, SS, NK, MK, JPF, and SO approved the final version of themanuscript.

## FUNDING INFORMATION

Funding provided by the Royal Society of New Zealand Te Apārangi Marsden Fund.

## CONFLICT OF INTEREST STATEMENT

No conflicts of interest, financial or otherwise, are declared by the authors.

## Data Availability

Study data are available from the corresponding author upon reasonable request.
